# Role of BDNF/ProBDNF Imbalance in Postoperative Cognitive Dysfunction by Modulating Synaptic Plasticity in Aged Mice

**DOI:** 10.3389/fnagi.2022.780972

**Published:** 2022-03-18

**Authors:** Ziyi Xue, Min Shui, Xiaowan Lin, Yi Sun, Jianhui Liu, Changwei Wei, Anshi Wu, Tianzuo Li

**Affiliations:** ^1^Department of Anesthesiology, Beijing Shijitan Hospital, Capital Medical University, Beijing, China; ^2^Department of Anesthesiology, Beijing Chao-Yang Hospital, Capital Medical University, Beijing, China; ^3^Department of Anesthesiology, School of Medicine, Tongji Hospital, Tongji University, Shanghai, China

**Keywords:** postoperative cognitive dysfunction, BDNF, ProBDNF, synaptic plasticity, aged

## Abstract

Postoperative cognitive dysfunction (POCD) is a disturbing neurological complication in patients undergoing anesthesia and surgical procedures. Brain-derived neurotrophic factor (BDNF) and its precursor proBDNF binding to their corresponding receptors tyrosine kinase (TrkB) and p75 neurotrophin receptor (p75NTR) exert quite an opposite biological function in neuron survival and synaptic function. This study aimed to demonstrate the critical role of the BDNF/proBDNF ratio in modulating synaptic plasticity, which further leads to anesthesia-/surgery-induced POCD. It also showed that the exogenous BDNF or p75NTR inhibitor could ameliorate cognitive dysfunction. In detail, 16-month-old C57BL/6 mice were subjected to a stabilized tibial fracture surgery with isoflurane anesthesia to establish the POCD animal model. The mice were then microinjected with either p75NTR inhibitor or exogenous BDNF into the dorsal hippocampus. Behavioral experiments were performed by open field and fear conditioning tests (FCTs). Western blotting was also used to measure the expression levels of BDNF, proBDNF, TrkB, p-TrkB, p75NTR, and synapse proteins. Golgi staining and electrophysiology were applied to evaluate the neuronal synaptic plasticity. Here, we demonstrated that anesthesia/surgery induced a reduction of BDNF/proBDNF, which negatively regulates the synaptic function in hippocampus, subsequently leading to cognitive impairment in aged mice. P75NTR inhibitor and exogenous BDNF could attenuate cognitive deficits by rescuing the dendritic spine loss and long-term potentiation (LTP) *via* altering the BDNF/proBDNF ratio. This study unveiled that the BDNF/proBDNF ratio in the hippocampus played a key role in anesthesia-/surgery-induced POCD. Thereby, tuning the ratio of BDNF/proBDNF is supposed to be a promising therapeutic target for POCD.

## Introduction

Postoperative cognitive dysfunction (POCD) is a common neurological complication in elderly surgical patients, which is mainly manifested clinically as declined learning, memory, concentration, and execution ability (Eckenhoff et al., 2020). Currently, clinical research revealed that POCD may contribute to adverse outcome, including increased morbidity and mortality, prolonged hospitalization, and reduced quality of life (Terrando et al., 2011). Since its mechanism stays unclear, whether anesthesia/surgery can be considered as etiologies of POCD remains controversial (Eckenhoff et al., 2020). At present, some acknowledged pathological mechanisms, including neuroinflammation (Safavynia and Goldstein, 2018), oxidative stress (Netto et al., 2018; Safavynia and Goldstein, 2018), Aβ protein metabolism (Shen et al., 2016), and neurotransmitter release disorders (Sarter et al., 2007), involved in POCD research were closely related to changes in synaptic plasticity. Synaptic plasticity, which can maintain neurons and neural circuits stability, plays an important role in learning, memory, cognitive function, and certain neurodegenerative diseases (Humeau and Choquet, 2019).

Brain-derived neurotrophic factor (BDNF) belongs to the neurotrophic family which is widely expressed in the central nervous system. BDNF exists in precursor and mature forms. ProBDNF can be secreted and proceeded extracellularly by plasmin or by matrix metalloproteases (MMPs) to produce mature BDNF (mBDNF) (Pang et al., 2004; Mizoguchi et al., 2011). Typically, tissue plasminogen activator (tPA) activated the extracellular protease plasmin to cleave proBDNF and produce mBDNF (also known as BDNF) (Pang et al., 2004). The main function of BDNF is to maintain neuronal survival and differentiation, facilitate synaptic transmission, enhance synaptic plasticity and strengthen synaptic growth through activation of TrkB receptor and ERK signaling cascade (Lu et al., 2013). However, its precursor, proBDNF, has quite the opposite biological properties, which is called a “Yin and Yang” model of neurotrophin action (Lu et al., 2005). ProBDNF causes cell death, dendritic arborization reduction, spine density loss (Yang et al., 2014), and long-term depression (LTD.) through its high-affinity p75 neurotrophin receptor (p75NTR) (Woo et al., 2005). Moreover, the combination of proBDNF binding with p75NTR will trigger the RhoGDI, which induces a reduction in spine density by modulating RhoA activity (Buhusi et al., 2017). TAT-Pep5, a peptide used to block the interaction between p75NTR and RhoGDI (Yamashita and Tohyama, 2003), has been verified to improve the behavior performance of elderly mice with memory impairment (Buhusi et al., 2017). Thus, regulating the ratio of extracellular proBDNF and BDNF seems to be crucial for synaptic function and memory consolidation.

Previous studies have shown that the reduction of BDNF is involved in the pathogenesis of surgery-induced POCD (Fan et al., 2016; Qiu et al., 2016). However, seldom research shows whether surgery can cause changes in proBDNF expression. Herein, we proposed a hypothesis that the ratio of BDNF/proBDNF by modulating synaptic plasticity plays a vital part in surgery-induced POCD in aged mice. Intrahippocampal infusions of TAT-Pep5 and exogenous administration of BDNF could attenuate surgery-induced cognitive deficits *via* tuning the ratio of BDNF/proBDNF and alleviate the deficiency of synaptic function, which is supposed to be a potential therapeutic target in future POCD prevention.

## Materials and Methods

### Animals

All experiments were approved by the Institutional Animal Care and Use Committee at Capital Medical University (Beijing, China) and carried out under the regulations of Medical Research Center of Capital Medical University (protocol AEEI-2020-117). Wild-type male C57BL/6 mice (16-month- old, weighing 28–32 g) were purchased from Beijing Vital River Laboratory Animal Technology Co., Ltd. (Beijing, China). Five mice per cage were housed (22–25°C) with access to food and water *ad libitum* under a 12-h light/dark cycle. All mice were adapted to the environment for 2 weeks and were randomly divided into treatment groups.

### Experimental Protocol

**FIGURE 1:**
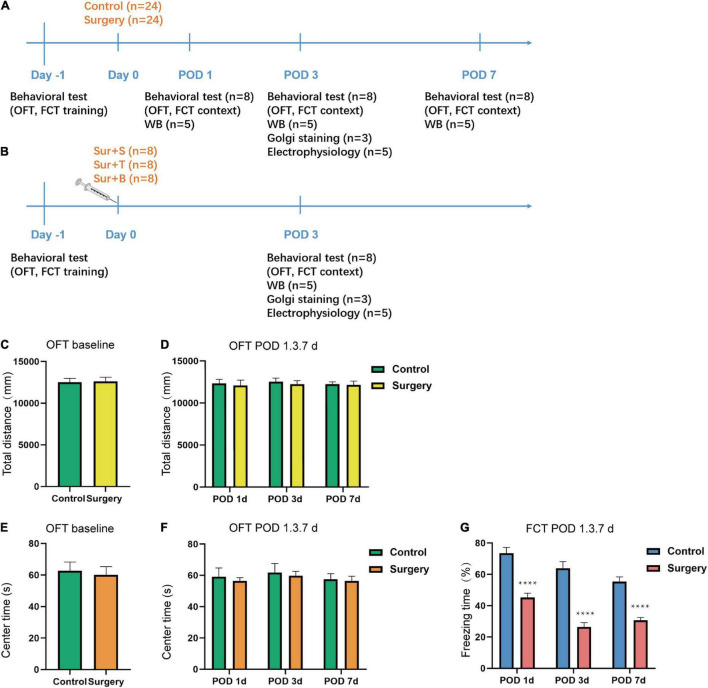
Schematic timeline of the experimental design and results of behavioral tests. **(A)** The mice received OFT and fear conditioning training on day 1. The OFT and fear conditioning context tests were performed on postoperative days 1, 3, 7 (POD1, 3, 7). Orthopedic surgery was performed on day 0. Hippocampal tissues were harvested at the end of the behavioral tests on POD1, 3, 7. Western blotting analysis of BDNF, proBDNF, TrkB, p-TrkB, p75NTR, PSD-95, SYP expression was performed on POD1, 3, 7. Golgi staining and electrophysiology were performed on POD3. **(B)** Saline, TAT-pep5, and BDNF were perfused into the CA1 subfield of the dorsal hippocampus followed by the orthopedic surgery on day 0 after the OFT and fear conditioning training (on day −1) and the fear conditioning context tests were performed on POD3. Western blotting analysis of BDNF, proBDNF, TrkB, p-TrkB, PSD-95, and SYP expression was performed on POD3. Golgi staining and electrophysiology were performed on POD3. **(C–F)** The OFT was conducted to assess the total distance and time spent in the center arena on day −1 and on POD1, 3, 7. **(G)** The percent of freezing time in the fear conditioning context test were assessed on POD1, 3, 7. Data are presented as the mean ± SEM (*n* = 24, OFT baseline; *n* = 8 OFT/FCT POD1, 3, 7). ****p < 0.0001 compared to the control group.

The whole experiment was composed into two parts. In the experiment section A, the mice were randomly sorted into 2 groups: the control group (C) and the surgery group (S). The S group (*n* = 24) received intramedullary fixation of tibial fractures under isoflurane anesthesia, while the C group (*n* = 24) received 100% oxygen without surgery/anesthesia. In the experiment section B, the mice were randomly divided into 3 groups: the surgery + saline (Sur + S, *n* = 8), the surgery + TAT-pep5 (Sur + T, *n* = 8), and surgery + BDNF (Sur + B, *n* = 8), and all the mice underwent orthopedic surgery. According to the fear conditioning results in section A, we chose postoperative day 3 (POD 3) for the time point of Golgi staining and LTP in section A. All the experiments in section B were implemented on POD 3. A schematic diagram of the experimental procedure is summarized in Figures 1A,B.

### Postoperative Cognitive Dysfunction Animal Model

Right tibial fracture with intramedullary fixation procedure was performed under anesthesia to establish the POCD animal model described in the previous study (Chen et al., 2019). Mice were subjected to 3% isoflurane for induction and 1.5% isoflurane for maintenance. Then an incision was made lateral to the right tibia to expose the bone and then a 0.38 mm intramedullary fixation needle was inserted into the tibial medullary canal for fixation. Finally, an osteotomy was performed in the middle and distal thirds of the tibia. The surgical incision was sutured with 4–0 silk non-absorbable suture (Mersilk; Ethicon, United States). The mice body temperature was adjusted at 36–37^°^C during the whole procedure by a heating pad. Ropivacaine (0.2%, Oxford; AstraZeneca) was applied locally to prevent postoperative pain.

### Drug Delivery Regimen

Mice were anesthetized with isoflurane mentioned above. A sufficient amount of drugs were injected into the CA1 subfield under the mouse brain atlas (AP ± 2.1 mm, ML ± 1.5 mm, DV −1.5 mm) through a bilateral stereotactic instrument. Soon after drug delivery, an orthopedic procedure was performed immediately. Mice in the group Sur + S were injected with saline 0.8 μl/side. Mice in the group Sur + T were subjected to a solution of TAT-Pep5 (EMD Millipore, Billerica, MA, United States) 20 ng/μl, 0.8 μl/side (Buhusi et al., 2017). Mice in the group Sur + B were administered BDNF (recombinant human BDNF,248-BDB, R&D systems, Emeryville, CA, United States) 0.25 μg/μl, 0.8 μl/side (Kim et al., 2012). All drug solutions were infused at a speed of 0.1 μl/min. The needle was retained for 5 min after the injection for drug absorption and then slowly withdrawn.

### Behavioral Tests

#### Open Field Test

We used the open field test (OFT) to assess the exploratory locomotor activity. According to the published protocol of OFT (Kraeuter et al., 2019), a white opaque plastic chamber (50 × 50 × 40 cm) was used as the open-field arena. The center zone was one-half of the total area. The mice were placed individually in the center of the area to acclimate the environment for 5 min and allowed to move freely in the square field for 5 min. The movement traces and parameters of the mice were recorded by the video camera automatically. The data were analyzed by the supporting ANY-maze software system (ANY-maze, Stoelting Co., IL, United States). We used the total distance to determine the locomotor activity of the mice, while the time spent in the central zone was used to access the anxiety level of the mice. After each test, the arena was cleaned with 75% alcohol to avoid olfactory cues.

#### Fear Conditioning Test

Fear conditioning test (FCT) has been widely used to assess hippocampal-dependent memory in POCD. We employed the protocol described in the previous study (Xiong et al., 2018). The FCT consisted of a training phase and a test phase. During the training session, the mice were placed in the chamber to adapt to the environment for 180 s. Next, a 20 s, 70 dB tone (conditional stimulus, CS), and 2 s and 0.75 mA foot shock (unconditional stimulus, US) were applied in two pairs (60 s interval). There was a 10- s interval between CS and US. After 30 s recovery, the mice were placed back into the cages. For the test session, we were only concerned about the hippocampus-dependent memory, so we performed the context test solely (Qiu et al., 2020). Mice were returned into the same chamber for 5 min without any stimulus on POD1,3, and 7. Freezing behavior was recorded and analyzed by the ANY-maze (ANY-maze, Stoelting Co., IL, United States) animal tracking system software. Likewise, to avoid the olfactory cues, 75% ethanol was used to clean the chamber.

### Western Blotting

Western blotting (WB) was performed as described before (Wang et al., 2020). Hippocampal total protein lysates were prepared by the RIPA lysis buffer (KeyGen Biotech, China) which contained the protease inhibitors and phosphatase inhibitors (Beyotime, China). Homogenized hippocampal tissues were quantified by Pierce BCA Protein Assay Kit (Thermo-Scientific, Waltham, MA, United States). Then, the denatured proteins were separated by 8 and 12% SDS-PAGE (Sigma-Aldrich) and transferred to 0.2 μm polyvinylidene difluoride (PVDF) membranes (Merck Millipore, ISEQ00010, United States). Membranes were blocked in a solution, including Tris-buffered saline with Tween (TBST), and 5% non-fat dry milk for 1 h at room temperature and incubated with primary antibodies overnight at 4°C. The primary antibodies were anti-TrkB (1:5,000, ab187041, United Kingdom), anti-phospho-TrkB (Y705) (anti-p-TrkB, 1:1,000, ab229908, United Kingdom), anti-p75NTR (1:1,000, 8238, Cell Signaling Technology, United States), anti-postsynaptic density protein 95 (anti-PSD95,1:1,000, 3450, Cell Signaling Technology, United States), anti-synaptophysin (anti-SYP, 1:1,000, 5461, Cell Signaling Technology, United States) and β-actin (1:5,000, 66009-1-Ig, proteintech, China). After evaluating several BDNF antibodies, anti-BDNF (1:1,000, ab108319, Abcam, United Kingdom) was chosen to reliably identify both BDNF (14 kDa) and proBDNF (34 kDa). After washing 3 times in TBST, the membranes were incubated for 1 h at room temperature with secondary antibodies (1:5,000, Gene-protein link, China). Blots were visualized using enhanced chemiluminescence (ECL) reagent (Millipore, Billerica, MA, United States) and imaged by VILBER Fusion FX6 Spectra (Vilber-Lourmat, Paris, France). For proteins with low expression and difficult to visualize, SuperSignal West Atto reagent (Thermo Fisher Scientific, Waltham, MA, United States) was used to detect them. The target protein bands were quantitated by Image J software (National Institutes of Health, Bethesda, MD, United States) and normalized to the density of β-actin in the same sample.

### Golgi-Cox Staining

Golgi-Cox staining was used to reveal the structure of dendritic spines in the hippocampus by using the Golgi Stain Kit (#PK401, FD NeuroTechnologies, Columbia, MD, United States). Mice brains were harvested and rinsed in the milli-Q water before being immersed in a mixture of solutions A and B for 3 weeks at room temperature in darkness. Then the brains were transferred to solution C for at least 72 h (up to 1 week). Next, the brains were cut into slices of 100 μm thickness with cryotome (CM3050 S, Leica, Wetzlar, Germany)(chamber temperature—23°C) and mounted on gelatin-coated microscope slides (#PO101, FD NeuroTechnologies, Columbia, MD, United States). Finally, slides were stained in a mixture of solutions D and E, dehydrated with alcohol, and cleared in xylene before mounting with a coverslip. The CA1 region of hippocampal neurons was captured by a Panoramic scan digital slice scanner (3DHISTECH Ltd., Budapest, Hungary). The spine densities were calculated from 2 independent dendritic spines derived from 3 separate images of each animal. The counting was performed by two experimenters independently. We used the Neuron J plugin in Fiji software (Fiji-win64, NIH, United States) to track the branches of dendrites and calculate the total length of dendrite branches. Sholl analysis (Sholl, 1953) was used to measure the intersection of dendrites in every 10 μm from the cell soma to reflex the complexity of neuronal dendrites.

### Electrophysiology

The 64-channel recording system (MED64, Panasonic Alpha-Med Sciences, Japan) was used to evaluate the function of synaptic plasticity by recording the long-term potentiation (LTP). By choosing one of the MED64 probes (Panasonic; MED-P515AP) as a stimulating electrode in the CA1 region of the hippocampus, the evoked field excitatory postsynaptic potentials (fEPSPs) were recorded by revised techniques described previously (Shimono et al., 2002) with a specially modified artificial cerebral spinal fluid (aCSF) formula for aging mice (Ting et al., 2014). Briefly, the mice were decapitated, and the brain tissue was placed in ice-cold NMDGaCSF filled with a gas mixture of 95% O_2_ and 5% CO_2_. Coronal slices (400 μm) were cut by a vibratome (Leica VT1200S) and incubated in NMDGaCSF at 32–34°C for no more than 12 min. Next, brain slices were transferred to a HEPES holding aCSF maintained at room temperature (22–25°C) for another 60 min prior to recording. Finally, the slices were placed in the MED probes and perfused with recording aCSF at a rate of 4 ml/min. Above all the steps, aCSF was needed to be continuously perfused with the mixed gas. LTP was induced by five episodes of theta burst stimulation (TBS) in accordance with the previous protocols (Zhang et al., 2017). FEPSP slopes and amplitudes (peak to peak) for each slice were derived from the data which were averaged by 5 consecutive probes in the CA1 region. Figures were presented as the percentages of mean fEPSP slopes and mean amplitude of the baseline (before TBS) period. We used the fEPSP slopes and amplitude over the last 10 min of the recording for statistical analysis.

### Statistical Analysis

Statistical analyses were performed with GraphPad Prism8.0 (GraphPad, San Diego, CA, United States.). Data are presented as mean ± S.E.M. In experiment section A, the difference of behavioral test and WB results were analyzed by one-way ANOVA with a Tukey *post-hoc* test for multiple comparisons. Total dendritic length, dendritic intensity, fEPSP slope, and amplitude were compared by unpaired *t*-test. The dendritic intersections were analyzed by two-way ANOVA. In experiment section B, three groups were analyzed by one-way ANOVA with a Tukey *post-hoc* test for multiple comparisons. The dendritic intersections were analyzed by two-way ANOVA with a Tukey *post-hoc* test for multiple comparisons. A significant difference was defined as *p* < 0.05.

## Results

### Anesthesia/Surgery Impaired Cognitive Function in Aged Mice

The OFT was applied to access the locomotor activity and anxiety-like behavior of aged mice. Stressful mice are showed less movement in the open area, while mice with lower anxiety are inclined to spend more time in the central area (Kraeuter et al., 2019). The result showed there was no significant difference in the total distance (*t* = 0.1364; *p* = 0.8921; Figure 1C) traveled by the mice and the time spent in the center arena (*t* = 0.1980; *p* = 0.7377; Figure 1E) between the C and S groups at baseline (Day −1). Additionally, there was no difference in the total distance (*t* = 0.3103, *p* = 0.7609, POD1; *t* = 0.4785, *p* = 0.6397, POD3; *t* = 0.1476, *p* = 0.8848, POD7; Figure 1D) and center time (*t* = 0.4561, *p* = 0.6553, POD1; *t* = 0.3062, *p* = 0.7639, POD3; *t* = 0.2123, *p* = 0.8349, POD7; Figure 1F) of the two groups on POD1, 3, 7. The fear conditioning contextual test was used to evaluate hippocampus-dependent long-term learning and memory. In the fear conditioning context test, the freezing time was significantly decreased on POD1, 3, and 7 in S group compared to the C group (POD1, *t* = 6.160; *p* < 0.0001; POD3, *t* = 7.354; *p* < 0.0001; POD7, *t* = 6.989; *p* < 0.0001; Figure 1G). Taken together, these results suggested that anesthesia/surgery-induced hippocampus-dependent memory declined while did not affect locomotor activity and anxiety-level in aged mice.

### Anesthesia/Surgery-Induced Hippocampal Deteriorated Synaptic Plasticity in Aged Mice With Postoperative Cognitive Dysfunction

**FIGURE 2:**
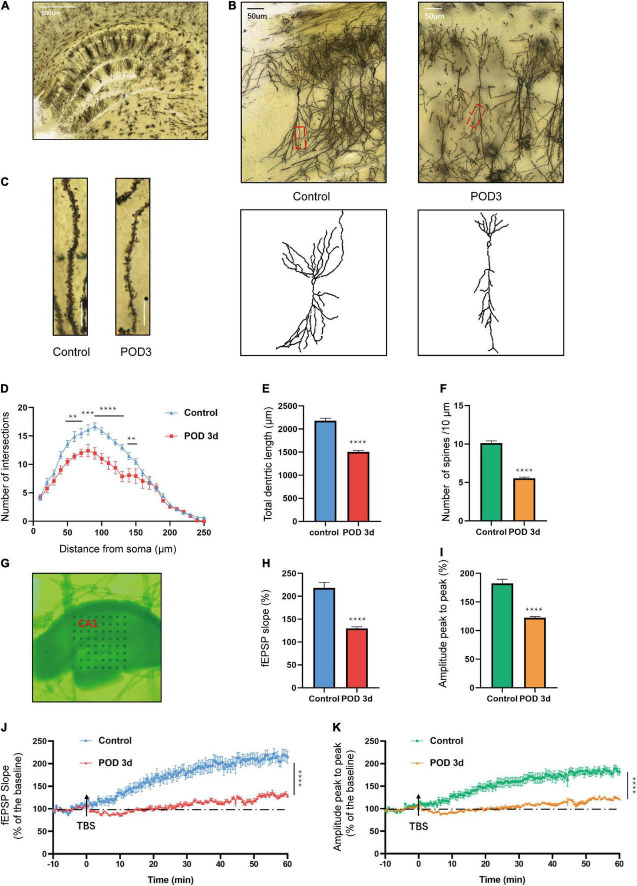
Hippocampal synaptic plasticity changes on POD3 of aged mice with POCD. **(A)** A dorsal hippocampal profile image of Golgi-Cox staining. Scale bar = 500 μm. **(B)** Sample image of Golgi-Cox staining and camera tracings of hippocampal CA1 neurons. Scale bar = 50 μm. **(C)** Representative dendritic spine density of hippocampal CA1 neurons. Scale bar = 10 μm. **(D)** Quantification of dendritic intersections (*n* = 18 from 3 mice). **(E)** Quantification of the total dendritic length (*n* = 18 from 3 mice). **(F)** Quantification of the spine density (*n* = 18 from 3 mice). **(G)** Sample image of the MED64 probes location in the CA1 region of dorsal hippocampus. **(H,I)** Average fEPSP slope/amplitude during the last 10 min. **(J,K)** LTP/amplitude recording in the hippocampal CA1 region. Data are the percentage of mean fEPSP slopes/amplitude recorded during the baseline period (*n* = 15 slices from 5 mice). Arrows show the time point of TBS delivery. Data are presented as the means ± SEM for each group. **p < 0.01 compared to the control group (***p < 0.001, ****p < 0.0001).

Previous studies have unveiled that hippocampal synaptic plasticity is a critical mechanism of memory formation, storage, and consolidation (Zhang et al., 2015). We used the Golgi-Cox staining to intuitively quantify the synaptic structure. The morphology of dorsal hippocampus and CA1 neurons are shown in Figures 2A–C. Sholl analysis was used to assess dendritic branching. The dendritic intersections at 50–140 μm from the soma were significantly declined in the S group [*F*_(24_, _350)_ = 4.151; *p* < 0.0001; Figure 2D]. Similarly, the total dendritic length reduced in the S group (*t* = 10.64; *p* < 0.0001; Figure 2E). Consistent with the above results, the spine density also declined in the S group (*t* = 13.60; *p* < 0.0001; Figure 2F). Additionally, the function of synaptic plasticity was measured by electrophysiology. MED64 systems were used to record the LTP in the CA1 region of hippocampus on POD 3 (Figure 2G). Average fEPSP slope (*t* = 7.073; *p* < 0.0001) and amplitude (*t* = 7.842; *p* < 0.0001) decreased obviously in the S group (Figures 2H,I). In the hippocampal slices of C group, LTP was induced steadily by TBS stimulation and recorded for at least 60 min. The amplitude (peak to peak) showed a similar result (Figures 2J,K). These results suggested that anesthesia/surgery exacerbated both morphology and function of synaptic plasticity in the hippocampal CA1 region of aged mice, which might be implied as an underlying mechanism of POCD.

### Anesthesia/Surgery-Induced Brain-Derived Neurotrophic Factor/Pro Brain-Derived Neurotrophic Factor Imbalance, Receptors, and Synaptic Proteins Changes in Aged Mice With Postoperative Cognitive Dysfunction

**FIGURE 3:**
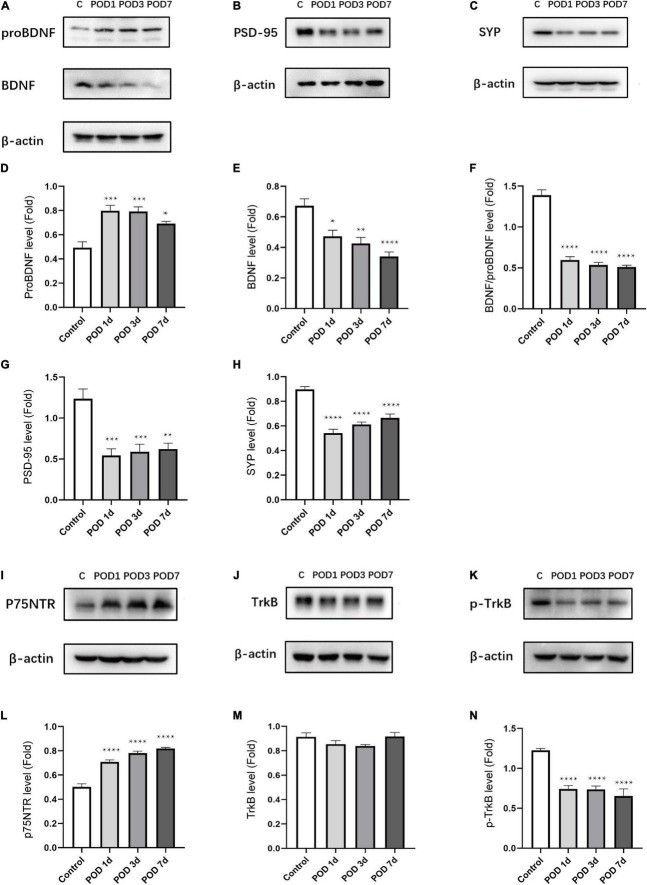
Anesthesia/surgery induced BDNF/proBDNF imbalance and related proteins changes in the hippocampus of aged mice. **(A–C)** Representative western blots of proBDNF, BDNF, PSD-95, SYP in hippocampus on POD1, 3 and 7. **(D–H)** Quantitative analysis of proBDNF, BDNF, BDNF/proBDNF, PSD-95, and SYP levels. **(I–K)** Representative western blots of p75NTR, TrkB, p-TrkB in hippocampus on POD1,3 and 7. **(L–N)** Quantitative analysis of p75NTR, TrkB, p-TrkB levels. Data are presented as the mean ± SEM (*n* = 5). *p < 0.05 compared to the control group (**p < 0.01, ***p < 0.001, ****p < 0.0001).

To determine whether anesthesia/surgery induced changes of BDNF/proBDNF expression in aged mice with POCD, we detected the proteins levels on POD 1, 3, and 7. Compared to C group, the proBDNF level was significantly increased [*F*_(3_, _16)_ = 12.96; *p* = 0.0001; Figures 3A,D] in the S group. In contrast, the BDNF level was significantly decreased [*F*_(3_, _16)_ = 13.08, *p* = 0.0001; Figures 3A,E] in S group. The ratio of BDNF and proBDNF was calculated to reflect the imbalance of the proteins intuitively. It turned out anesthesia/surgery significantly downregulate the BDNF/proBDNF levels [*F*_(3_, _16)_ = 93.88; *p* < 0.0001; Figure 3F]. To evaluate the effects on synapse quantity, we measured the presynaptic protein SYP and postsynaptic protein PSD-95 level in the hippocampus. The level of PSD-95 declined compared to C group [*F*_(3_, _16)_ = 12.40; *p* = 0.0002; Figures 3B,G]. Consistent with PSD-95, SYP expression declined simultaneously [*F*_(3_, _16)_ = 32.86; *p* < 0.0001; Figures 3C,H].

To determine whether anesthesia/surgery impacts the corresponding receptors expression, we measured the expression level of p75NTR and TrkB. The p75NTR level was obviously upregulated in comparison with the C group [*F*_(3_, _16)_ = 64.73; *p* < 0.0001; Figures 3I,L]. By binding with BDNF, the activation form of TrkB (p-TrkB) can lead to downstream signaling proteins activation. We found that there was no significant difference of TrkB between the C and S groups [*F*_(3_, _16)_ = 1.969; *p* = 0.1594; Figures 3J,M]. However, the p-TrkB level was significantly downregulated in comparison with the C group [*F*_(3_, _16)_ = 22.29; *p* < 0.0001; Figures 3K,N].

These results suggested that anesthesia/surgery downregulated BDNF and p-TrkB activation as well as synaptic protein (PSD-95, SYP) levels in the hippocampus. Correspondingly, the proBDNF and p75NTR were upregulated conversely. These indicated the role of BDNF/proBDNF imbalance played an important role in surgery-induced POCD in age mice.

### P75NTR Inhibitor and Exogenous Brain-Derived Neurotrophic Factor Rescued Brain-Derived Neurotrophic Factor/Pro Brain-Derived Neurotrophic Factor Imbalance, Receptors, and Synaptic Proteins Expression in Aged Mice With Postoperative Cognitive Dysfunction

**FIGURE 4:**
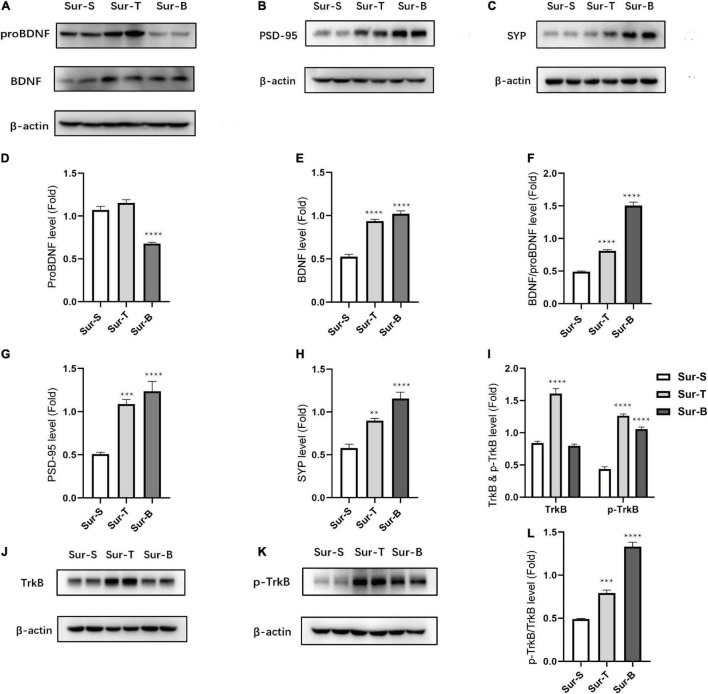
Inhibition of p75NTR and exogenous BDNF rescued ratio imbalance and related proteins changes in hippocampus of aged mice with POCD. **(A–C)** Representative western blots of proBDNF, BDNF, PSD-95, and SYP in hippocampus on POD3. **(D–H)** Quantitative analysis of proBDNF, BDNF, BDNF/proBDNF, PSD-95, and SYP levels. **(J,K)** Representative western blots of TrkB, p-TrkB in hippocampus on POD3. **(I–L)** Quantitative analysis of p-TrkB/TrkB, TrkB, p-TrkB levels. Data are presented as the mean ± SEM (*n* = 5). **p < 0.01 compared to the control group (***p < 0.001, ****p < 0.0001).

To determine the effect of p75NTR inhibitor and exogenous administration of BDNF on the imbalance of BDNF/proBDNF expression in aged mice with POCD, we detected the protein levels on POD3. The levels of proBDNF significantly decreased in Sur-B group, while there was no statistic difference between Sur-T group and Sur-S group [*F*_(2_, _12)_ = 55.37, *p* < 0.0001; Sur-S vs. Sur-B, *p* < 0.0001; Sur-S vs. Sur-T, *p* = 0.2542; Figures 4A,D]. In stark contrast, the levels of BDNF significantly increased in Sur-T group and Sur-B group [*F*_(2_, _12)_ = 87.97; *p* < 0.0001; Figures 4A,E]. We used the ratios to determine whether TAT-pep5 and exogenous BDNF rescued the imbalance of BDNF/proBDNF levels. It turned out these two interferences could upregulate the ratios of BDNF and proBDNF [*F*_(2_, _12)_ = 239.3; *p* < 0.0001; Figure 4F].

Receptor activation and synaptic protein expression were consistent with the ratio alternation of BDNF/proBDNF. The PSD-95 level increased in the Sur-T group and Sur-B group [*F*_(2_, _12)_ = 26.61, *p* < 0.0001; Sur-S vs. Sur-T, *p* = 0.0004; Sur-S vs. Sur-B, *p* < 0.0001; Figures 4B,G]. The SYP level also improved obviously in Sur-T group and Sur-B group [*F*_(2_, _12)_ = 29.42, *p* < 0.0001; Sur-S vs. Sur-T, *p* = 0.003; Sur-S vs. Sur-B, *p* < 0.0001; Figures 4C,H]. We used the p-TrkB/TrkB ratio to determine the receptor activation (Figures 4I–K). P-TrkB/TrkB level increased in the Sur-T group and Sur-B group [*F*_(2_, _12)_ = 141.2, *p* < 0.0001; Sur-S vs. Sur-T,*p* = 0.0002; Sur-S vs. Sur-B, *p* < 0.0001; Figure 4L].

These results indicated that p75NTR inhibitor (TAT-pep5) would not affect the expression level of proBDNF while its beneficial effect on POCD could somehow upregulate the expression level of BDNF. In the meantime, exogenous administration of BDNF could downregulate the proBDNF level accordingly. Both of them lead to ratio alternation, which ameliorates cognitive impairment in aged mice.

### P75NTR Inhibitor and Exogenous Brain-Derived Neurotrophic Factor Improved Synaptic Plasticity and Alleviated Anesthesia/Surgery-Induced Memory Loss in Aged Mice With Postoperative Cognitive Dysfunction

**FIGURE 5:**
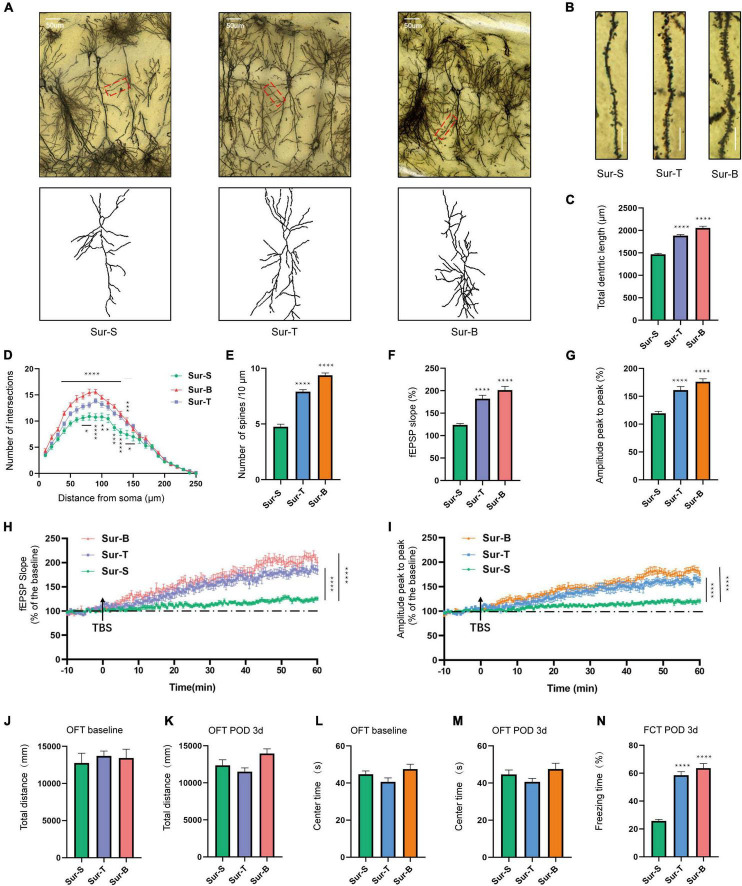
Inhibition of p75NTR and exogenous BDNF ameliorated hippocampal synaptic plasticity impairment and facilitate memory consolidation of aged mice with POCD. **(A)** Sample image of Golgi-Cox staining and camera tracings of hippocampal CA1 neurons among the 3 groups. Scale bar = 50 μm. **(B)** Representative dendritic spine density of hippocampal CA1 neurons. Scale bar = 10 μm. **(C)** Quantification of the total dendritic length (*n* = 18 from 3 mice). **(D)** Quantification of dendritic intersections (*n* = 18 from 3 mice). **(E)** Quantification of the spine density (*n* = 18 from 3 mice). **(F,G)** Average fEPSP slope/amplitude during the last 10 min. **(H,I)** LTP/amplitude recording in the hippocampal CA1 region. Data are the percentage of mean fEPSP slopes/amplitude recorded during the baseline period (*n* = 15 slices from 5 mice). Arrows show the time point of TBS delivery. **(J–M)** The total distance and time spent in the center arena on day −1 and on POD1, 3, 7. **(N)** The percent of freezing time in the FCT context was assessed on POD3. Data are presented as the mean ± SEM (*n* = 8) *p < 0.05 compared to the control group (**p < 0.01, ***p < 0.001, ****p < 0.0001).

The morphology of dorsal hippocampus and CA1 neurons among 3 groups is shown in Figures 5A,B. In terms of synaptic structural plasticity, TAT-pep5, and exogenous BDNF significantly upregulated the total dendritic length in Sur-T and Sur-B groups compared to the Sur-S group [*F*_(2_, _51)_ = 107.5; *p* < 0.0001; Figure 5C]. Sholl analysis showed that the total number of dendritic intersections at 80—90,130 μm from the soma improved in Sur-T group [*F*_(24_, _350)_ = 3.015; *p* < 0.0001; Figure 5D]. Likewise, the number of dendritic intersections at 50–130 μm from the soma were largely improved in the Sur-B group [*F*_(24_, _350)_ = 7.303; *p* < 0.0001; Figure 5D]. Similarly, the spine density in Sur-T and Sur-B groups apparently increased in comparison to the Sur-S group [*F*_(2_, _51)_ = 132.7; *p* < 0.0001; Figure 5E]. In terms of synaptic functional plasticity, hippocampal LTP in CA1 region was evaluated on POD3. As it turned out, p75 inhibitor and exogenous BDNF alleviated the LTP and amplitude loss in Sur-S group. Average fEPSP [*F*_(2_, _42)_ = 36.12; *p* < 0.0001] and amplitude [*F*_(2_, _42)_ = 33.13; *p* < 0.0001] improved largely compared to the Sur-S group (Figures 5F,G). In the hippocampal slices, LTP in Sur-T and Sur-B groups earned an obvious promotion compared to the Sur-S group, same as the amplitude (Figures 5H,I). These results suggested p75 inhibitor and extraneous BDNF may alleviate surgery-induced synaptic plasticity dysfunction, which attenuated cognitive impairment in aged mice.

In the OFT, the total distance [*F*_(2_, _21)_ = 0.2013; *p* = 0.8193] and center time [*F*_(2_, _21)_ = 0.2439; *p* = 0.7858] were not significantly different among the 3 groups at baseline (Figures 5J,L) or on POD3 [total distance *F*_(2_, _21)_ = 3.655; *p* = 0.0435; center time *F*_(2_, _21)_ = 2.412; *p* = 0.1140; Figures 5K,M]. These results showed that drug delivery regimen or the orthopedic operation did not affect the locomotor ability of mice. Moreover, p75 inhibitor and exogenous BDNF increased the freezing time in Sur-T and Sur-B groups compared to the Sur-S group in the context test [*F*_(2_, _21)_ = 68.75; *p* < 0.0001; Figure 5N]. To sum up, these results indicated that inhibition of p75NTR and exogenous BDNF rescued hippocampus-dependent cognitive decline induced by anesthesia/surgery in aged mice.

## Discussion

The purpose of the current study showed that the balance of BDNF and proBDNF played a critical role in anesthesia/surgery-induced POCD in aged mice. We found that the expression of BDNF was largely decreased under surgical trauma with anesthesia in aged mice with POCD, while the proBDNF level was increased. In addition, anesthesia/surgery exerted a detrimental effect on synaptic function which gives us a glimpse of the correlation between the imbalanced BDNF/proBDNF expression and synaptic plasticity in hippocampus of POCD mice. Moreover, the p75NTR inhibitor and the exogenous BDNF could rescue surgery-induced imbalance of BDNF and its precursor, and establish a relative new ratio homeostasis, which further ameliorates the synaptic dysfunction and cognitive impairment. Hence, these results indicated the vital role of BDNF/proBDNF in the therapeutic cognitive dysfunction induced by anesthesia/surgery in aged mice.

Although the pathological mechanism of POCD is complex and intricate, there is accumulating evidence suggesting the correlation between deficits of synaptic plasticity and POCD. For instance, surgical trauma could activate the hippocampal complement C3, causing a decrease in the expression of pre- and post-synaptic proteins SYP and PSD-95, which contributed to cognitive decline (Xiong et al., 2018). Moreover, surgical trauma could disrupt PGE2-EP3 signaling pathway by reducing hippocampal synaptic plasticity-related proteins, such as CREB, BDNF, and Arc, which participate in the formation of LTP (Xiao et al., 2018). Anesthesia/surgery led to mammalian target of rapamycin (mTOR) hyperactivity in the hippocampus and interfered with synaptic proteins homeostasis by inhibiting autophagy and resulted in cognitive impairment (Gao et al., 2021). Early pathological stage of POCD created an environment of inflammation and stimulated A1-specific astrocyte responses ultimately leading to sustained synaptic inhibition and cognitive deficiency (Li et al., 2020). In our study, we proved that anesthesia/surgery-induced an abnormal conversion of BDNF and its precursor (proBDNF), breaking the stable balance of the ratio in hippocampus of aged mice and consequently, leading to POCD *via* modulating the synaptic plasticity.

The ratio of BDNF/proBDNF varies between particular stages of brain development and regions. Pro-BDNF is highly concentrated in the postnatal period and drops gradually while BDNF expresses throughout postnatal development and prevails in adulthood (Yang et al., 2009, 2014). Efficient conversion to BDNF in adulthood is pivotal for its beneficial function in neuronal synaptic plasticity (Kowiański et al., 2018). As for the aging period, the study indicated that proBDNF and p75NTR increased largely in aged mice. Though the BDNF level has not shown any statistical difference, tPA, plasmin, and p-TrkB/TrkB declined accompanied with aging (Buhusi et al., 2017). These results fully illustrate in aged mice, proBDNF/BDNF increases (partly attributes to the reduction level of tPA and plasmin) and negatively regulates synaptic remodeling in coordinate with p75NTR. P75NTR inhibitor could rescue the synaptic deficits and memory consolidation. In our study, anesthesia/surgery not only induced reduction in BDNF level but also increment in proBDNF and p75NTR levels that have not been verified in other studies. The opposite changes down-regulated BDNF/proBDNF and led to dendritic spine shrinkage and declined in LTP. We also detected the rescue effect of the p75NTR inhibitor has not shown a significant impact on the proBDNF expression, while its beneficial impact on spine density, synapse proteins, and LTP promotion could feedback upregulate BDNF level and increase the BDNF/proBDNF ratio. We speculate that TAT-pep5 may influence the conversion of proBDNF to BDNF by reducing the formation of other fragments and facilitating more BDNF formation. It is known that the binding of proBDNF to p75NTR will trigger the association between p75NTR and RhoGDI, which eventually results in the reduction in spine density by modulating RhoA activity (Yamashita and Tohyama, 2003; Koshimizu et al., 2009), and RhoA activation is also essential in LTP induction by means of cofilin phosphorylation and inactivation (Rex et al., 2009). These results indicated that TAT-pep5, which blocked the interaction between p75NTR and RhoGDI, could largely improve synaptic plasticity by modulating BDNF/proBDNF. Thus, p75NTR could be considered as a potential therapeutic target for aging-related memory deficiency.

The binding of BDNF to TrkB will initiate a positive signal to enhance synaptic transmission and facilitate LTP induction and dendritic spine growth. However, proBDNF associated with p75NTR will act as a negative signal to accelerate neuron death, synaptic degeneration, and LTD (Barker, 2009). Previous studies suggested anesthesia/surgery-induced the activation of hippocampal microglia, the release of IL-1β, and the reduction of BDNF, which led to the cognitive deficiency in elderly mice (Qiu et al., 2016). Research also suggested BDNF expression and phosphorylation/activation of TrkB and ERK declined after surgery, while the expression of total TrkB was not significantly affected (Fan et al., 2016). The latest study also suggested that anesthesia/surgery-induced reduction of BDNF expression, and the study proposed that neuroinflammation and overactivated calcium signaling pathway may facilitate these changes. It was also suggested the expression of total TrkB was significantly decreased (Qiu et al., 2020) which contradicts with the study mentioned above. We reckon this discordance may attribute to the different surgical types, age of mice, and time point of tissue harvesting. In our study, we also found that the anesthesia/surgery reduced the expression of BDNF and p-TrkB, while the levels of total TrkB not statistically decreased. It is shown in other work that the anesthesia/surgery-induced spine density loss (Qiu et al., 2020; Liu et al., 2021), LTP decrease (Liu et al., 2021), and synapse proteins reduction (Xiong et al., 2018), which were consistent with previous research. To further understand the roles of BDNF and proBDNF in the development of POCD, exogenous BDNF and p75 inhibitor were microinjected into the mice hippocampus. The BDNF level increased as expected after being injected with exogenous BDNF, however, it is interesting that the proBDNF level decreased. We speculate the extra BDNF can suppress the proBDNF expression built on a negative feedback regulation mechanism. In this study, we also found that exogenous BDNF can promote the formation of dendritic spine arborization and LTP, which largely prospered the synaptic plasticity in the cognitively declined mice. The behavioral test shows that the anesthesia/surgery impaired hippocampus-dependent memory consolidation, which is consistent with the previous findings (Fan et al., 2016; Xiong et al., 2018; Qiu et al., 2020; Liu et al., 2021). In the FCT, context learning is associated with dorsal hippocampus, especially in the CA3 region. During the training session, we used the pattern of trace conditioning (interval or gap between US and CS) to isolate hippocampal involvement (Curzon et al., 2009). In accordance with the upregulation of BDNF/proBDNF by p75NTR inhibitor and exogenous BDNF, the memory decline has been largely improved. Taken together, these data indicated that alternation of BDNF/proBDNF may be an efficient method in the therapy of POCD.

The mechanism of how the imbalanced BDNF/proBDNF occurred in the current study deserved deeper thinking. Neuroinflammation is considered to be involved with several signaling pathways in correlation with BDNF. Nuclear factor-kappa B (NF-κB) is a transcription factor as well as the main factor of inflammatory activator, which can induce the expression of pro- and anti-apoptotic genes, including BDNF (Lima Giacobbo et al., 2019). Surgery-induced neuroinflammation triggered iron imbalance, microglia activation, and BDNF impairments, while deferoxamine (a clinically used iron chelator) improved memory impairment by ameliorating inflammation and upregulating BDNF expression (Li et al., 2016). BDNF-pathway has been indicated as a mediator between neuroinflammation and cognitive impairment, and also IL-1β played a crucial role in regulation of BDNF levels (Hovens et al., 2014). So we suppose that the abnormal expression of BDNF/proBDNF level may be largely due to the inflammatory in anesthesia/surgery-induced brain disorders which remained further testified in our future study.

Accumulating evidence suggested that BDNF/proBDNF imbalance also impacts a variety of neurological diseases aside from cognitive decline. BDNF/proBDNF in hippocampus is critical in CUMS-induced depressive-like behaviors by alternating dendritic spines in the hippocampal CA1 pyramidal neurons (Qiao et al., 2017). BDNF/proBDNF is also crucial in the pathogenesis of post-stroke depression (PSD) in ischemic hippocampus. Aerobic exercise could relieve depression symptoms, promote neurogenesis, and increase the ratio of BDNF/proBDNF in the ischemic hippocampus of PSD rats (Luo et al., 2019). BDNF and proBDNF and their associated proteins participated in the pathogenesis and recovered from photothrombotic ischemia (Rahman et al., 2018). Our study indicates the ratio of BDNF/proBDNF is a key role in POCD in aged mice by modulating synaptic plasticity, which may open up new avenues in the research of neurocognitive diseases.

There are some limitations in this study. First, only short-term (7 days) cognitive performance by FCT after anesthesia and surgery was evaluated. It is expected that the detection of the effect on medium term (1–3 months), as well as the spatial learning and memory measured by Morris water maze or Barnes maze experiments should be investigated in a future study. Second, an anesthesia control group was not set up since we reckoned general anesthesia and surgical procedure were undividable in clinical practice. Studies revealed that inhaled anesthetic was also a key element involved in the POCD pathogenesis (Ni et al., 2015; Chen et al., 2020), so we would use sevoflurane as the inhaled anesthetic in the further study, which is consistent with the clinical procedures. Third, a C group was not established in experiment section B. since we considered the procedure of stereotactic brain injection itself as a minor craniocerebral surgery, so the Sur + S was used as the baseline. In the future study, a C + saline group (without orthopedic surgery) is expected to be set up for receiving a more convincing conclusion. Forth, there is a lack of explanation of the obvious upregulation of TrkB level in Sur-T. Alternatively, we used p-TrkB/TrkB to evaluate the signal activation degree in different groups. It is supposed such phenomenon might be derived from a feedback regulation intertwined with other receptors, though intensive research is needed to be further performed.

## Conclusion

In summary, our study demonstrated that anesthesia/surgery-induced the imbalance of BDNF/proBDNF expression in the hippocampus and deteriorated neuronal synaptic plasticity. The broken homeostasis may ultimately cause cognitive impairment and memory decline. The result of this study suggests a novel hint in rescuing cognitive impairment by regulating the BDNF/proBDNF ratio. The TAT-pep5 (p75 inhibitor) and the exogenous BDNF can upregulate the ratio, which may be a potential therapeutic treatment of anesthesia/surgery-induced cognitive impairment.

## Data Availability Statement

The original contributions presented in the study are included in the article/supplementary material, further inquiries can be directed to the corresponding author/s.

## Ethics Statement

The animal study was reviewed and approved by all procedures were approved by the Institutional Animal Care and Use Committee at Capital Medical University (protocol AEEI-2020-117).

## Author Contributions

CW and ZX designed the research. ZX, MS, and XL performed the research. MS, YS, and JL analyzed the data. ZX contributed to writing-original draft preparation. CW, AW, and TL contributed to writing, review, and editing, supervision, funding acquisition. All authors approved the final manuscript.

## Conflict of Interest

The authors declare that the research was conducted in the absence of any commercial or financial relationships that could be construed as a potential conflict of interest.

## Publisher’s Note

All claims expressed in this article are solely those of the authors and do not necessarily represent those of their affiliated organizations, or those of the publisher, the editors and the reviewers. Any product that may be evaluated in this article, or claim that may be made by its manufacturer, is not guaranteed or endorsed by the publisher.
